# Navigating Postpartum Venous Thromboembolism: A Case of Thrombophilia, Bleeding Complications, and Chronic Inferior Vena Cava Syndrome

**DOI:** 10.7759/cureus.71753

**Published:** 2024-10-18

**Authors:** Bianka Stancheva, Bistra Boneva, Mario Stankev, Detelina Lukanova

**Affiliations:** 1 Vascular Surgery, National Cardiology Hospital, Sofia, BGR; 2 Angiology, National Heart Hospital, Sofia, BGR

**Keywords:** inferior vena cava syndrome, postpartum venous thromboembolism, thrombophilia, uterine bleeding, venous stenting

## Abstract

Postpartum venous thromboembolism (VTE), encompassing deep vein thrombosis (DVT) and pulmonary embolism (PE), is a critical complication occurring in the postpartum period. The pathogenesis involves a hypercoagulable state induced by pregnancy-related physiological changes, venous stasis from reduced mobility and pelvic compression during delivery, and endothelial injury. Postpartum VTE is a leading cause of maternal morbidity and mortality, necessitating heightened clinical vigilance. Understanding the risk factors, implementing prophylactic measures, and ensuring timely intervention are paramount for improving maternal health outcomes related to venous embolic events.

The presented case is a 37-year-old female with a complex medical history marked by recurrent thrombotic events and pregnancy complications. Despite various prophylactic and therapeutic interventions, her condition culminated in severe chronic venous obstruction (CVO) requiring advanced interventional treatment and stent-graft implantation. Her medical history began in 2012 with two spontaneous abortions, leading to the identification of genetic mutations, including a homozygous methylenetetrahydrofolate reductase (MTHFR) mutation. In 2016, she developed PE after receiving hormonal contraceptive therapy without antithrombotic prophylaxis. Subsequent pregnancy was closely monitored, yet she suffered severe complications, including a cesarean delivery complicated by preeclampsia and postoperative thrombocytopenia, leading to massive iliofemoral-popliteal DVT. Initial treatment with vitamin K antagonists (VKA) was replaced with apixaban following a recurrent thrombotic event. Despite optimal anticoagulation, the patient developed symptomatic inferior vena cava (IVC) syndrome in 2022, characterized by chronic IVC occlusion, acute thrombosis of the portal and inferior mesenteric veins, and extensive collateral venous networks. She underwent recanalization and stenting of the iliac veins and IVC. This was followed by a hysterectomy due to metrorrhagia, significantly improving her quality of life.

In this case, the homozygous MTHFR mutation was associated with recurrent thrombotic events and pregnancy complications. Despite multiple guidelines advising against MTHFR testing for thrombosis evaluation, the patient's management was influenced by her genetic profile and clinical history. Direct oral anticoagulants (DOACs) have shown efficacy in treating VTE in patients with hereditary thrombophilia. The clinical case also highlights the complexity of anticoagulation management, particularly after venous stenting, where maintaining stent patency poses significant challenges. Venous stenting, especially in CVO, has demonstrated substantial benefits, as evidenced by the patient's marked symptom improvement post-procedure. The long-term efficacy of stenting, optimal anticoagulation strategies, and post-procedural therapy require further research. This case highlights the complexities of managing hereditary thrombophilia with recurrent thrombosis and the evolving role of venous stenting in treating CVO. Individualized anticoagulation and multidisciplinary care are essential, with further studies needed to refine treatment and improve outcomes.

## Introduction

Pregnancy and the postpartum period are associated with significant physiological changes that create a hypercoagulable state. This prothrombotic state increases the risk of deep vein thrombosis (DVT) and pulmonary embolism (PE). Several mechanisms contribute to this increased risk:

Hormonal and hematological changes

The levels of the sex hormones estrogen and progesterone increase during pregnancy, leading to the enhanced production of coagulation factors, especially fibrinogen and factors VII, VIII, IX, and X. This creates a propensity for clot formation. On the other hand, the activity of fibrinolytic proteins (like tissue plasminogen activator) is reduced [1]. There is a relative decrease in natural anticoagulants (like protein S and antithrombin III), tipping the balance towards coagulation. Platelets become more reactive during pregnancy, further contributing to the prothrombotic state [2]. 

Hemodynamic changes

The growing uterus exerts pressure on the inferior vena cava (IVC) and pelvic veins, reducing venous return from the lower extremities and causing venous stasis, which could be a critical factor in thrombus formation. Blood volume expands by approximately 30-50% during pregnancy, which can lead to venous dilation and stasis [3]. In pregnant women, most thrombotic events occur in the left iliofemoral and IVC because of the increased venous stasis and compression by the pregnant uterus [4,5].

Physiological adaptations

The body's mechanisms to ensure rapid and effective clot formation in response to injury (such as during childbirth) are heightened, but this can also predispose to pathological clot formation [1]. In other words, there is an enhanced clot stability.

The postpartum period, particularly the first six weeks after delivery, is associated with an even higher risk of thromboembolic events [3]. Delivery trauma during vaginal delivery or cesarean section leads to increased tissue factor release, which also promotes coagulation. Postpartum recovery often involves periods of immobility, further contributing to venous stasis. Although hormone levels begin to normalize postpartum, the prothrombotic effects can persist, maintaining the elevated risk for thromboembolic events. Additionally, the postpartum period involves healing and inflammatory processes that can activate endothelial cells lining the blood vessels, promoting clot formation. Due to these factors, the incidence of DVT and PE increases significantly during pregnancy and the postpartum period. Studies have shown that the risk of venous thromboembolism (VTE) is approximately 5-10 times higher in pregnant women compared to non-pregnant women of the same age [6]. This risk is highest in the postpartum period, where it is estimated to be about 20-fold higher than in non-pregnant women [7]. Given the heightened risk, it is crucial for healthcare providers to take specific measures to manage and mitigate the risk of thromboembolic events in postpartum women. This includes identifying high-risk patients, such as those with a history of thrombosis, thrombophilia, obesity, prolonged immobility, or other risk factors, for closer monitoring. Prophylactic measures, like anticoagulation with low-molecular-weight heparin (LMWH), may be considered during pregnancy and the postpartum period for these high-risk individuals [6,8]. Additionally, encouraging early mobilization postpartum can help reduce venous stasis. Educating patients about the signs and symptoms of DVT and PE, such as leg pain or swelling and sudden shortness of breath, respectively, is also vital for timely intervention.

In addition to the general physiological changes during pregnancy and the postpartum period that create a hypercoagulable state, women with inherited thrombophilia face an even greater risk of thromboembolic events. Inherited thrombophilias are genetic disorders that increase the likelihood of abnormal blood clot formation. Common types include factor V Leiden mutation, prothrombin G20210A mutation, protein C and protein S deficiency, and antithrombin III deficiency​ [9].

The increased risk of thromboembolic events in women with inherited thrombophilia during pregnancy and the postpartum period underscores the need for vigilant monitoring and management. The hypercoagulable state, already heightened by pregnancy, is exacerbated by these genetic disorders, making the postpartum period particularly critical. One of the serious consequences of this heightened risk is DVT, which, if not promptly addressed, can lead to significant complications such as chronic venous disease (CVD). This, in turn, can progress to chronic vena cava syndrome, a condition that severely impacts a patient's quality of life due to persistent venous obstruction and associated symptoms like chronic edema, pain, venous hypertension, and ulceration. Early intervention is essential to mitigate these risks, prevent further morbidity, and improve long-term outcomes.

## Case presentation

We present the case of a young female patient, who has faced a complex medical journey marked by significant thrombotic events and pregnancy complications. The medical challenges began in 2012, at the age of 26, following two spontaneous abortions. A DNA analysis conducted at that time revealed the presence of genetic mutations, homozygous genotype methylenetetrahydrofolate reductase (MTHFR), heterozygous genotype vascular endothelial growth factor A (VEGFA), and heterozygous haplotype annexin A5 (ANXA5), with only the MTHFR mutation being directly associated with VTE. 

In 2016, after being prescribed hormonal contraceptive therapy without antithrombotic prophylaxis due to amenorrhea, the patient developed a PE and was treated with vitamin K antagonists (VKA) for four months. In 2019, during a well-monitored pregnancy that followed all prophylactic guidelines with LMWH in adequate doses, the patient experienced a complicated cesarean delivery due to preeclampsia, which was further complicated by severe postoperative thrombocytopenia. Two days postoperatively, she was diagnosed with massive iliofemoral-popliteal DVT in the left lower limb and was subsequently hospitalized in our vascular surgery department. After five days of therapeutic dosage of LMWH, VKA (acenocoumarol) therapy was reintroduced.

Despite optimal control of the international normalized ratio (INR), she experienced a spontaneous thrombotic event in a superficial vein of the upper limb several months later. Consequently, VKA was replaced with apixaban at a therapeutic dose of 5 mg twice daily due to the assumption of a more stable plasma concentration of the drug, allowing for adequate anticoagulation, which can sometimes be difficult to achieve and unpredictable with the use of VKA. A year later, complete recanalization of the affected deep veins in the left lower limb was confirmed via ultrasound, and the treatment was adjusted to a prophylactic reduced dose of apixaban 2.5 mg twice daily.

**Figure 1:**
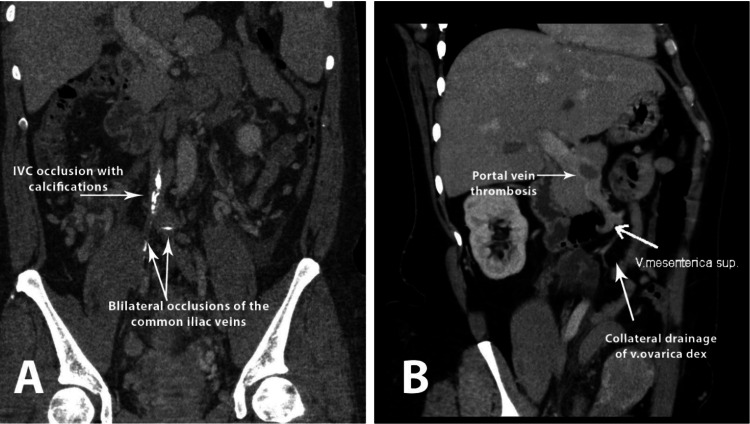
CT venography prior to the interventional treatment revealed chronic occlusion (A) IVC with calcifications and occlusion of the iliac and renal veins bilaterally. (B) Occlusion of the left ovarian vein and acute thrombosis of the portal vein and inferior mesenteric vein IVC: inferior vena cava

By the end of 2022, the patient began to complain of shortness of breath, general fatigue, and pelvic pain. Closer clinical examination suspected IVC syndrome, including bilateral lower limb heaviness, edema, and pelvic and lower extremity pain. Notably, there was the presence of dilated superficial veins on the abdominal wall, indicative of collateral circulation due to impaired venous return. These symptoms suggested significant venous obstruction, likely leading to increased venous pressure and compromised venous outflow. A CT venography was performed and revealed symptomatic IVC syndrome (Figure 1) as follows: chronic occlusion of the IVC with calcifications along its course, occlusion of the iliac and renal veins bilaterally, as well as the left ovarian vein, and acute thrombosis of the portal vein and inferior mesenteric vein. The inferior mesenteric vein was dilated to 21 mm, tortuous, and unenhanced, while the superior mesenteric vein remained patent and dilated to 11 mm, facilitating venous drainage from the lower limbs through a rich collateral network. Numerous collateral venous vessels were also identified (Figure 1).

The anticoagulant was returned to a therapeutic dose, with several vascular specialists recommending continued conservative treatment. Additionally, the patient reported intermittent vaginal bleeding, which had been exacerbated by an increased dosage of anticoagulant therapy.

**Figure 2:**
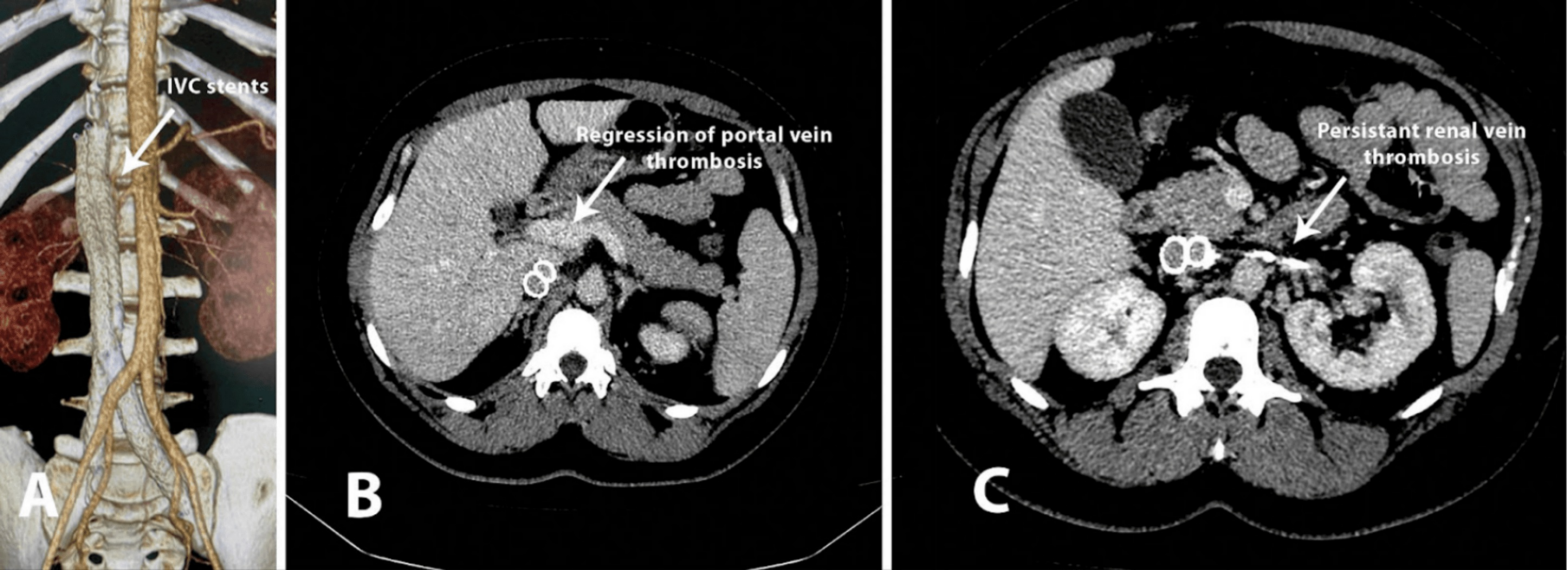
Postoperative CT (A) 3D reconstruction of the patent ilio-caval stents. (B) Regression of the thrombosis in the portal vein. (C) Persistent occlusion of the renal veins IVC: inferior vena cava

Seeking definitive treatment for her condition, the patient underwent recanalization and stenting of the iliac veins and the IVC in another hospital, three months later. The procedure was performed via bilateral femoral and right axillary access. The occlusion of vascular cognitive impairment (VCI) was passed through axillary access in an antegrade manner. Kissing stents were implanted in VCI and iliac veins bilaterally (Venovo, Bard, New Providence, NJ, USA), 14/120 mm and 16/120 mm for the left side and 12/100 mm and 14/100 mm for the right side. She was discharged on a regimen of rivaroxaban 20 mg and aspirin 100 mg daily. Post-stenting CT imaging one month after the procedure showed the regression of the thrombosis in the portal vein, mild reduction in subcutaneous venous collaterals, persistent occlusion of the renal veins with renal drainage occurring through an extensive collateral network, and formation of cavo-portal shunts behind the right kidney. The venous stents were patent, and a congestive periuterine venous plexus was observed (Figure 2). Four months later, a hysterectomy was performed due to the ongoing metrorrhagia of varying intensities and durations since 2020, which had significantly impacted her quality of life while on mandatory anticoagulant therapy.

All of the above lead to significant clinical improvement in the patient's condition and a complete reduction of their symptoms.

## Discussion

Thrombophilia promotes a prothrombotic state through elevated procoagulant factors, reduced anticoagulants, and proinflammatory or autoimmune mechanisms. Hereditary thrombophilia is categorized as mild or severe based on the thrombotic risk associated with specific genetic anomalies. Some hereditary anomalies in anticoagulant mechanisms, such as antithrombin deficiency, protein C and S deficiencies, and factor V Leiden mutation, are established risk factors for VTE [10].

Decisions regarding testing for hereditary thrombophilia are made on a case-by-case basis, contributing to the long-term treatment plan [11]. During pregnancy, normal changes occur in the coagulation system, such as increases in coagulation factors Vc, VIIIc, and Xc and von Willebrand factor antigen, along with decreases in total and free protein S. These physiological changes result in a relative thrombotic tendency. Among the mutations identified, our patient is only homozygous for the MTHFR mutation.

The MTHFR (C677T) mutation results in reduced gene activity, leading to the decreased synthesis of 5-methyltetrahydrofolate, the primary methyl donor in converting homocysteine to methionine. Consequently, elevated plasma homocysteine levels (hyperhomocysteinemia) occur, which is a recognized risk factor for thrombosis. The most common acquired cause of this condition remains dietary deficiency of folate and vitamin B12. The homozygous MTHFR mutation is present in 1-4% of the general population and is associated with a threefold increased risk of DVT and PE, preeclampsia, and placental abruption. Despite all of the above-mentioned current guidelines from professional societies, such as the American College of Medical Genetics [12], American College of Obstetricians and Gynecologists, British Society for Haematology, Society for Maternal‐Fetal Medicine, and Choosing Wisely initiative of the American Board of Internal Medicine, all agree that MTHFR testing should not be performed for the evaluation of thrombosis. Eliminating MTHFR from thrombophilia testing will reduce patient concerns and healthcare costs [13]. 

However, it is crucial to recognize that for our patient, knowing about the underlying thrombophilia significantly impacts her clinical management. The presence of mutations associated with thrombosis complicates her pregnancy, posing risks for both maternal and fetal health, including the potential development of VTE and chronic IVC occlusion. Each mutation contributes to her overall thrombotic risk profile, which necessitates tailored monitoring and intervention strategies during her prenatal care. In certain cases, dismissing the relevance of MTHFR mutation testing may overlook potential risk factors that could adversely affect maternal outcomes. Thus, we advocate for a nuanced approach that considers individual patient history and genetic findings, emphasizing the need for personalized care despite the generalized guidelines.

When evaluating a patient with inherited thrombophilia, it is important to consider other coexisting risk factors, whether genetic or acquired, that may contribute to the risk of first or recurrent VTE. This assessment should guide decisions on the necessity and duration of primary prophylaxis. Prevention strategies should be individualized based on the patient's specific situation and risk factors.

The gold standard for preventing spontaneous abortion and other obstetric complications in such cases is the administration of low-dose aspirin or LMWH. The Cochrane Collaboration database reports a 15% reduction in preeclampsia and a 14% reduction in fetal and/or neonatal mortality with this prophylaxis. The reduction in mortality is most pronounced in high-risk women. The combination of aspirin and LMWH is effective in recurrent pregnancy loss in antiphospholipid syndrome and may be considered for women with hereditary thrombophilias and a history of severe preeclampsia, placental abruption, intrauterine growth restriction, or fetal loss, although controlled studies on this topic are currently lacking [14].

In the case presented, the patient was administered LMWH during her pregnancy, in accordance with international guidelines. Despite this appropriate pharmacological management, she experienced complications, including thrombocytopenia and preeclampsia, which necessitated an emergency cesarean section. Following this surgical intervention, the patient developed DVT. This case underscores that while LMWH is regarded as the gold standard for preventing obstetric complications, there are instances where severe adverse events can still occur, highlighting the need for continuous monitoring and personalized treatment approaches for high-risk patients.

A brief highlight of a significant meta-analysis that included 10 clinical trials involving patients with VTE and underlying thrombophilia demonstrated that direct oral anticoagulants (DOACs) are both safe and effective for treating VTE and preventing recurrence in patients with thrombophilia, regardless of the specific genetic mutation constellation [15]. However, DOACs are not recommended for patients with a history of thrombosis who are diagnosed with antiphospholipid syndrome, particularly those who are triple-positive (for lupus anticoagulant, anticardiolipin antibodies, and anti-beta 2 glycoprotein I antibodies) and thus at an increased risk of recurrence. When choosing an anticoagulant, factors such as patient preferences, adherence to therapy, the nature of previous thrombotic events, difficulty in controlling INR, and quality of life should be carefully evaluated in patients with thrombophilia.

Anticoagulants are often necessary for preventing or treating thromboembolic events in postpartum women, including those with inherited thrombophilia. When it comes to breastfeeding, certain anticoagulants are considered safe for use. Warfarin, for example, does not pass into breast milk in significant amounts and is generally safe for breastfeeding mothers. LMWH like enoxaparin and dalteparin are also preferred because they have large molecular structures that limit their transfer into breast milk, making them safe for the infant. However, DOACs like rivaroxaban and apixaban are less well studied in breastfeeding and are generally not recommended due to the potential risk of transfer into breast milk. Therefore, when anticoagulation therapy is necessary during breastfeeding, the choice of anticoagulant should be carefully considered to ensure both maternal health and infant safety [15].

The presented clinical case involves the initiation of treatment with a VKA at a therapeutic dose, based on the aforementioned considerations. Following the occurrence of a thrombotic complication with a different location, it was replaced with therapeutic-dose apixaban, and after ultrasound-verified recanalization of the deep veins, the patient transitioned to prophylactic long-term therapy. Despite all the adhered recommendations, the patient developed chronic occlusion of the IVC, with corresponding symptoms that compromise her quality of life.

The management of chronic venous obstruction (CVO) often requires a multidisciplinary approach, including considerations for potential interventions to restore venous patency, as well as ongoing symptom management and supportive care. Follow-up evaluations are critical to monitor the progression of the condition and to adjust the treatment strategy accordingly.

Given the complexity of the case, it is essential to continue reassessing the patient's clinical status, considering the balance between the benefits and risks of anticoagulation therapy, and exploring additional treatment modalities as needed to improve overall patient outcomes.

Let's delve into the minimally invasive, patient-preferred interventional treatment methods and the specific indications for their use in this particular pathology. The indications for venous stenting are as follows: symptomatic CVO which includes conditions such as May-Thurner syndrome, CVO in patients with chronic venous insufficiency, post-thrombotic CVO (affecting the upper or lower limbs), and pelvic venous disorders in the presence of CVO [16]. Special cases include renal vein compression (nutcracker syndrome), chronic occlusions of the IVC, adolescent and pediatric patients, and splanchnic venous disorders. 

The signs and symptoms of CVO are diverse, ranging from pain, cramps, heaviness, paresthesia, pruritus, edema, skin induration and hyperpigmentation, ulceration, vulvar varicosity, lower limb varicosity, pelvic pain, dyspareunia, hematuria, and proteinuria. Our patient presented with a significant number of these complaints.

Upon reviewing the diagnostic and treatment algorithm for CVO as outlined in the Cardiovascular and Interventional Radiological Society of Europe (CIRSE) guidelines, and applying it to this case, it is clear that our patient meets the criteria for venous stenting. The patient presents with symptoms consistent with CVD [16], and the ilio-caval occlusion has been confirmed through CT venography. Based on this, the patient falls within the Clinical-Etiology-Anatomy-Pathophysiology (CEAP) Classification stage C3-6, which justifies the recommendation for recanalization and venous stenting. This clinical stage indicates significant venous pathology, where intervention is indicated to alleviate symptoms and prevent further progression*.*

By definition, a clinically significant obstruction is present when there is a pressure gradient of 2-3 mmHg across the lesion in a supine patient. However, this measurement is practically impossible to obtain. Another potential indicator is a 50% reduction in the cross-sectional diameter compared to a reference vein, a criterion frequently used by physicians, though it has not been validated by clinical studies. The presence of collateral vessels is another consideration. Many physicians believe that the presence of collaterals indicates significant obstruction, while the absence suggests the opposite. However, since most patients are examined in a supine position, it is challenging to accurately assess the collateral network, making it a potentially misleading marker. Lately, the intravascular imaging modality has been a preferred tool for the preprocedural assessment of the severity of venous obstruction with its high sensitivity. It is essential not only for diagnosis but also in the process of accurate stent placement. Over time, intravascular ultrasound (IVUS) has become a crucial imaging technique for better evaluating the structure of the vessel wall and identifying intra-luminal issues like changes in mural thickness, synechiae, spurs, trabeculations, frozen valves, and external compressions that are not always visible with traditional venous angiography. Furthermore, IVUS provides precise measurements of venous stenosis. Today, IVUS is widely regarded as the gold standard imaging method, and its use is recommended for evaluating symptomatic patients with suspected venous outflow obstruction [17].

In one of the largest single-center retrospective studies, 528 limbs with reflux in the deep venous system were stented in the iliac segment. The five-year results showed an 88% patency rate, a 54% healing rate for venous ulcers, a 78% improvement in pain symptoms, and a 55% improvement in edema [18]. 

In 2015, results from a randomized double-blind study that compared stenting plus medical therapy versus medical therapy alone were published. The primary endpoints were changes in the Visual Analog Scale (VAS) score and the rate of venous ulcer closure over six months. As the results indicated, stent implantation led to a dramatic improvement in pain symptoms, with statistically significant differences between the two groups, as well as a higher rate of venous ulcer closure. Additionally, there was a significant improvement in quality of life, as assessed by the relevant questionnaire [19]. 

In summary, for patients with chronic venous insufficiency and proximal obstruction, stenting leads to an improvement in the patient's clinical condition. These outcomes are confirmed by the presented clinical case, as the patient currently has no complaints related to her lower limbs.

A significant meta-analysis from 2015 reviewed over 30 clinical studies, examining the technical success of interventions based on the underlying pathology, whether non-thrombotic, acute thrombotic, or chronic post-thrombotic. It also assessed the complications that arose from these procedures. The meta-analysis revealed a very low complication rate alongside impressive percentages of technical success. Predictably, technical difficulties were more common in cases involving chronically occluded venous vessels. The highest five-year patency rates were observed in non-thrombotic cases, while the lowest were in chronic post-thrombotic occlusions. This outcome was expected because these patients typically suffer from poor inflow and have long segments of damaged veins. The secondary patency rate evaluated in the meta-analysis was also very good and commendable. Therefore, venous stenting rightfully deserves its place in our arsenal of treatment strategies [20]. 

As observed, the clinical outcomes for patients who undergo venous stenting are excellent in cases of acute thrombosis and good in non-thrombotic lesions, and around 70% of patients with chronic occlusions experience significant clinical improvement in their symptoms [20]. The presented clinical case falls into the third group, dealing with a chronic occlusion. Remarkably, the patient's response to treatment exceeded expectations, with a marked reduction in symptoms and a substantial improvement in overall quality of life, highlighting the effectiveness of venous stenting even in challenging cases of chronic occlusion.

Deep venous stenting is being increasingly utilized, thanks to the development of specialized venous stents. One of the biggest challenges remains maintaining stent patency. Currently, there is no evidence to suggest that one anticoagulant is superior in preventing post-procedural stent thrombosis. Therefore, the choice of anticoagulant, and its combination with antiplatelet therapy, is largely individualized. Our patient was discharged on a combination factor Xa inhibitor and antiplatelet therapy after the interventional procedure. According to a small study of nine patients, a combination of rivaroxaban and tailored antiplatelet therapy (clopidogrel) is both safe and effective in maintaining vessel patency following endovascular stenting for iliofemoral post-thrombotic venous obstruction, with no occurrences of restenosis, stent occlusion, or significant bleeding observed during the follow-up period [21]. Future research will need to focus on determining the optimal duration of treatment and secondary prophylaxis following stenting. Nevertheless, the anticoagulation strategy will likely need to be tailored to the individual patient. Until more data is available, it is prudent to follow up with all patients and intervene if necessary.

For instance, in a particular case, a patient presented with intermittent episodes of metrorrhagia following delivery, which were notably exacerbated by the administration of anticoagulant therapy. This highlights the critical importance of individualized treatment plans and close monitoring in patients receiving anticoagulation. After undergoing venous stenting which reduced her overall symptoms but required long-term anticoagulant prophylaxis, the abnormal uterine bleeding persisted. After careful consideration of her reproductive goals and having an already healthy child, the patient was elected to undergo a hysterectomy as a definitive treatment to address the ongoing bleeding and improve her quality of life. Several studies suggest a consistent increased risk of severe uterine bleeding with rivaroxaban compared with VKA, enoxaparin, and apixaban [22,23].

## Conclusions

The presented case underscores the complexities of managing thromboembolic events in the context of pregnancy and postpartum, particularly in patients with inherited thrombophilia. Despite appropriate prophylactic measures, such as the use of LMWH during pregnancy, the patient experienced significant complications, including massive iliofemoral-popliteal DVT and chronic IVC occlusion. These complications illustrate the limitations of current standard treatments and the necessity for individualized and vigilant monitoring of high-risk patients. The successful recanalization and stenting of the iliac veins and IVC, along with subsequent clinical improvement, demonstrate the potential of minimally invasive interventional treatments in managing CVO. It is too early, from an evidence-based perspective, to assert that every symptomatic central venous obstruction is an indication for stenting, although it is very likely that patients will experience clinical improvement post-stenting. Post-procedural therapy still raises many unanswered questions, and the patient should be considered in their entirety by a multidisciplinary team.

In conclusion, the management of thromboembolic events in pregnancy and the postpartum period, particularly in patients with inherited thrombophilia, requires a personalized approach that balances the risks and benefits of anticoagulation therapy and interventional procedures. Ongoing research and clinical trials are crucial to further refine treatment protocols and improve long-term outcomes for these patients.
